# The Effects of Quinoa and Amaranth Flour on the Qualitative Characteristics of Gluten-Free Cakes

**DOI:** 10.1155/2023/6042636

**Published:** 2023-06-14

**Authors:** Razieh Hamzehpour, Asiye Ahmadi Dastgerdi

**Affiliations:** Department of Food Science and Technology, Ardestan Branch, Islamic Azad University, Ardestan, Iran

## Abstract

The effect of pseudocereal flour such as quinoa and amaranth in different concentrations (0, 10, 20, and 30%) was investigated in gluten-free cake formulation. Cake containing amaranth and quinoa flour showed higher protein, fat, ash, and fiber content. A30 (30% amaranth) and Q30 (30% quinoa) had the highest values, and the control sample had the lowest values. The moisture content of the gluten-free cakes was not in the range of the Iranian standard. The sample containing 20% quinoa flour showed the highest specific volume (2.88 ± 0.09 cm^3^/g) and the lowest hardness (259.33 ± 10.09 g) (*p* < 0.05). *L*^∗^ value varied from 72.09 to 79, and the gluten-free cakes had a darker color. All the cakes containing quinoa and amaranth flour showed low *a*^∗^ and *b*^∗^ values (*p* < 0.05). Gluten-free cakes contain high amounts of unsaturated fatty acids (linoleic and linolenic acids) and minerals (iron, calcium, magnesium, and zinc). The results of the sensory evaluation showed that the sample containing 10% of amaranth and quinoa flour obtained the highest taste, aroma, color, appearance, and overall acceptability score compared to other treatments. In conclusion, it is possible to produce gluten-free cakes with quinoa and amaranth flour with sensory and physicochemical properties similar to wheat cakes. The formulations containing 20% and 30% quinoa were the best.

## 1. Introduction

Celiac is a common disease of the digestive system, which is also known as gluten-sensitive enteropathy. The immune system of patients responds by destroying the tissue of the small intestine, especially in the villi of the intestine, which absorb nutrients. Symptoms of this disease include nutrient absorption disorders, weight loss, diarrhea, anemia, fatigue, iron deficiency, and osteoporosis [1]. The only treatment for celiac is to avoid gluten-containing foods. Therefore, the foods containing gluten should be eliminated from the diet of patients [2].

Gluten-free products are usually made from pseudocereal flour and are not as nutritionally rich as gluten-containing products. They have several technical problems, including unfavorable texture, low specific volume, rapid staleness, poor color, and poor flavor [3]. The gluten-free dough has less strength and stretchability and high adhesion. All these factors significantly affect the ability of the dough to trap gas, low specific volume, brittle texture, poor color, and reduced shelf life [4].

Quinoa (*Chenopodium quinoa*) is a pseudocereal that originated in Latin America and is currently being consumed in Europe and around the world. It improves the nutritional quality of bakery products due to the proper balance of carbohydrates, proteins, lipids, minerals, and bioactive compounds [5, 6]. Quinoa not only increases the protein content but can also improve the biological value of the proteins [7]. It can also increase the content of unsaturated fatty acids and improve the ratio of omega-3/omega-6 fatty acids [8]. Quinoa has a high proportion of dietary fiber and reduces cholesterol levels as a source of prebiotics [9].

Amaranth (*Amaranthus hypochondriacus*) had higher nutritional quality than cereal grains such as high protein content and balanced essential amino acid composition. Amaranth protein is rich in lysine, usually lacking in cereals [10, 11]. It is characterized by a high fiber, fat content, and minerals, especially calcium and magnesium. Amaranth contains some antinutritional agents such as trypsin inhibitors, phytic acid, and phytate. Phytate content in amaranth ranges from 4.8 to 9.4 mmol/g. Trypsin inhibitors are at low levels that do not pose a risk to nutritional status [12].

Quinoa and amaranth can partially replace wheat flour in bread and increase its nutritional value, dietary fiber, minerals, high biological value proteins, and fats. Bread enriched with quinoa and amaranth is more effective as a functional food. Due to the nutritional composition of quinoa and amaranth, many studies investigated their effect on the quality properties of bread [3–8].

The aim of this research was to investigate the effect of quinoa and amaranth flour on the physicochemical, nutritional, and sensory properties of gluten-free cake.

## 2. Material and Methods

### 2.1. Materials

Wheat flour was purchased from Atlas Company (Isfahan, Iran). Quinoa and amaranth seeds were obtained from a store, and after grinding, the flour was sieved with 5 *μ*m. Salt from Sepiddane Company (Shiraz, Iran), sugar from Naqsh Jahan Company (Isfahan, Iran), and oil from Bahar Company (Iran) were procured. All chemicals were obtained from Merck (Germany).

### 2.2. Flour Composition

The wheat, quinoa, and amaranth flour were characterized for moisture content (AACC, 44-16), ash (AACC, 08-01), fiber (AACC, 32-10), fat (AACC, 10-30), and protein (AACC, 46-12) [13].

### 2.3. Cake Production

Quinoa and amaranth flour were mixed with wheat flour with replacement ratios of 0, 10, 20, and 30%. Sugar (30%) and eggs (40%) were mixed and stirred for 5 min. After mixing the oil (8%) and baking powder (3%), the cake was baked at 170°C for 20 min. The samples were cooled and packed in polyethylene bags and kept at room temperature for analysis. The cake prepared from 100% wheat flour was evaluated as a control sample.

### 2.4. Proximate Determination

The moisture, fat, protein, fiber, and ash contents were determined according to AACC [13] methods, respectively.

### 2.5. Physical Characteristics

The specific volume was obtained according to AACC method [13]. Texture was analyzed with texture analyzer (Brookfield, USA) with a probe with a diameter of 16 mm. The force was set to 500 N, and the probe speed was set to 20 mm/min. The maximum force was calculated as hardness [14, 15]. The color parameters, i.e., *L*, *a*^∗^, and *b*^∗^ values, were evaluated using HunterLab (FMS Jansen GmbH & Co.KG, USA) [16, 17].

### 2.6. Nutritional Characteristics

After the preparation of fatty acid methyl ester, gas chromatography (Agilent 7890A, USA) with FID detector was used to analyze fatty acids. For methylation, 50 *μ*L of sample was mixed with 100 *μ*L of 0.5 sodium methoxide, and 1 mL of hexane and methylation was carried out for 15 min at room temperature. The hexane layer, which also contains oil, after being separated from the aqueous solution, was poured into a container containing anhydrous sodium sulfate to remove the moisture, and the dehydrated sample was injected into the GC. The method of injection into GC was manual. The minerals were detected by atomic absorption spectroscopy (Spectro ARCOS, Germany) equipped with ADAX database, background correction, and cathode lamps [5]. All samples were wet mineralized with a mixture of acids: nitric and perchloric (3 : 1).

### 2.7. Sensory Evaluations

Color, flavor, appearance, odor, and overall acceptability were evaluated by nontrained 20 panelists using the 5-point hedonic scale. The samples were coded before the test.

### 2.8. Statistical Analysis

All experiments were conducted in a completely randomized design and in three replications. The results were expressed as mean ± standard deviation (SD). Analysis of variance (ANOVA) was done by SPSS 20 software (*p* < 0.05).

## 3. Results and Discussion

### 3.1. Proximate Composition of Flour

The chemical composition of the used flours is shown in Table 1. The moisture, protein, fat, ash, and fiber contents were higher in amaranth and quinoa flour than the wheat flour (*p* < 0.05).

The protein content of quinoa and amaranth flours was consistent with previous reports: 13.1-21.5%, 8-22%, and 18.2-25.3% [10, 18, 19]. The protein content of quinoa is similar to that of wheat and oats and higher than that of corn, rice, and barley. Protein content and amino acid profile change depending on growth conditions and genotype [20]. Amaranth and quinoa are rich sources of minerals and fiber [12, 21, 22]. The fiber amount of amaranth is relatively higher compared to common grains such as corn and wheat, which explains its higher levels of protein and fat content [11].

### 3.2. Proximate Composition of the Formulated Cake

The chemical characteristics of gluten-free cakes are presented in Table 2. The gluten-free cakes formulated with pseudocereal flour showed the higher ash, fat, protein, and fiber contents than the control sample (*p* < 0.05).

Moisture content is one of the desirable indicators in a cake, and this feature eventually makes the cake soft. The moisture level of the gluten-free cakes was not in the range of the Iranian national standard [23]. It can be said that the presence of hygroscopic compounds such as fiber in the structure of amaranth and quinoa flour caused an increase in the moisture content of the final product. The high fiber content prevents water evaporation during cooking and increases the moisture content [10, 14, 15, 22]. Some reports indicate that bread prepared from bran has high moisture content and low hardness during storage, which is attributed to the high capacity of bran in moisture retention [24]. Therefore, the presence of amaranth and quinoa flour in the cake formulation leads to an increase in the moisture content of the samples due to the increase in the amount of fiber.

According to the findings of Sanz-Penella et al. [12] and Miranda-Ramos et al. [10], the amaranth flour increases the protein, fat, ash, and fiber contents of bread. Cotovanu and Mironeasa [25] showed that amaranth flour led to a significant increase in protein, fat, and ash contents, while the moisture content decreased. Franco et al. [26] and Iglesias-Puig et al. [21] showed similar results with the addition of quinoa flour to gluten-free bread formulation.

### 3.3. Physical Characteristics of Cake

The physical characteristics of gluten-free cakes are presented in Table 3.

#### 3.3.1. Specific Volume

Specific volume is important for acceptance by consumers because cakes with higher specific volumes are usually preferred [17]. All the cake samples showed a specific volume from 2.30 to 2.88 cm^3^/g (Table 3). The sample containing 20% quinoa flour presented the highest specific volume (*p* < 0.05).

Quinoa has low amylase activity, which may lead to increased gas production and thus increased cake volume [27]. Also, the increased specific volume is probably due to the presence of high fiber content and hydrocolloids in amaranth and quinoa flour, which increased the water binding of flour and increased the specific volume [10, 22]. There is a possibility that the reduction of the specific volume in high amounts of amaranth and quinoa flour substitution is caused by the excessive increase in the thickness of the air cells in the dough that it will not have the ability to expand and increase the volume at oven [15, 21].

#### 3.3.2. Hardness

The results for the hardness of the cake ranged from 259.33 to 568.97 g (Table 3). The sample containing 20% and 10% quinoa flour showed the lowest and highest hardness (*p* < 0.05), respectively.

The hardness reduction in 20% quinoa cake is due to the adjustment of the gluten network and a porous texture and the preservation of moisture due to the high amount of fiber [10, 14, 15], while increasing the hardness in the higher level of amaranth and quinoa flour is due to the excessive absorption of water and a dense texture. The gluten decreased with the replacement of amaranth and quinoa flour and induced a loose network, weakening the dough structure and increasing the hardness [15, 24]. Also, it seems that the fat and hydrocolloids in amaranth and quinoa flour were not able to compensate for the hardness caused by gluten reduction [28]. Amaranth and quinoa flours contain a higher amount of fat that traps air bubbles and thus creates porosity that increases the hardness [14].

Sanz-Penella et al. [12], Nasir et al. [14], and Cotovanu and Mironeasa [25] showed that amaranth flour increases the hardness and elasticity of bread. Xu et al. [15] showed that higher amounts of quinoa flour (10% and 15%) increased the hardness of bread. Franco et al. [26] showed that quinoa bread had poor textural characteristics. A direct relationship between hardness and volume has been reported [15, 29], which is consistent with the results of this research.

#### 3.3.3. Color

The color analysis showed that *L*^∗^ parameter ranged from 72.09 to 79.09 (Table 3). Gluten-free cakes were characterized by a darker color. This can be attributed to the presence of fiber compounds in quinoa and amaranth flours and the moisture retention ability [9, 24]. The samples containing quinoa and amaranth flour were identified with higher values for *a*^∗^ and *b*^∗^ values; therefore, these samples were strongly red and yellow.

The difference in color parameters can be due to natural pigments in amaranth and quinoa flour. Carotenoids, chlorophyll, and lignin affect the color of the flour and the final product [27]. The color of the cakes is affected by the Maillard reaction and caramelization. Low substitutions of the amaranth and quinoa flour are effective on color changes due to the role of water-binding, and at high substitutions, the role of its pigments increases [12, 22].

The average values found for *L*^∗^, *a*^∗^, and *b*^∗^ values in the present study are similar to those found for the other gluten-free products containing amaranth and quinoa [14, 16, 17].

### 3.4. Nutritional Characteristics of Cake

According to the results presented in Table 4, cake samples containing amaranth and quinoa have more unsaturated fatty acids (C 18 : 1, C 18 : 2, and C 18 : 3) and minerals (Fe, Ca, Mn, and Zn) compared to the control cake.

Amaranth and quinoa increase nutritional value of cereal products [12, 21]. Ballester-Sánchez et al. [5, 6] found that quinoa improves polyunsaturated fatty acids (linoleic and linolenic acids) and dietary fiber in bread and improves the iron and zinc contents. El-Sohaimy et al. [30] showed that magnesium, calcium, iron, and zinc contents of quinoa bread were more than the wheat bread. Iglesias-Puig et al. [21] showed that quinoa increased the nutritional value of bread such as minerals (calcium, iron, and zinc).

### 3.5. Sensory Characteristics of Cake

**Figure 1:**
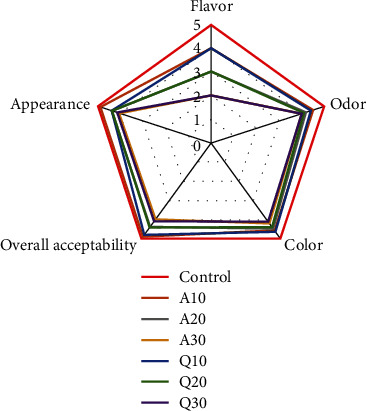
Sensory analysis of the gluten-free cake. A10: sample containing 10% amaranth flour; A20: sample containing 20% amaranth flour; A30: sample containing 30% amaranth flour; Q10: sample containing 10% quinoa flour; A20: sample containing 20% quinoa flour; A30: sample containing 30% quinoa flour.

Figure 1 shows the sensory characteristics of the formulated gluten-free cake samples. The control sample was the most acceptable to the consumer (*p* < 0.05). Among the gluten-free samples, the sample containing 10% of amaranth and quinoa flour had the highest scores for taste, aroma, color, appearance, and overall acceptance, due to the high ability of the fiber to preserve moisture during cooking [10, 14]. Samples containing 30% amaranth and quinoa flour got the lowest scores (*p* < 0.05), due to the insufficient cohesiveness of the gluten network, the appearance of the taste, odor, and color of amaranth and quinoa flour [15, 24].

In the research of Nasir et al. [14], the bread prepared by substituting 5% and 10% of amaranth flour is acceptable. Natalia et al. [24] showed that amaranth bread was close to traditional samples. Xu et al. [15] showed that the taste of bread increased significantly with the addition of quinoa flour. El-Sohaimy et al. [30] showed that most of the participants accepted and preferred bread with quinoa flour more than the control. Azizi et al. [3] showed that gluten-free bread with quinoa flour has favorable properties and the highest sensory score related to 25% quinoa flour.

## 4. Conclusion

Cake samples containing amaranth and quinoa showed higher protein, lipid, ash, and fiber contents, which improve their nutritional profile. Gluten-free cake showed high specific volume, low hardness, and good color compared to the control sample. This research opens up new opportunities for the gluten-free bakery industry and demonstrates the possibility of producing gluten-free products for a group of populations with special needs, such as celiacs, gluten intolerant, diabetics, and/or both.

## Figures and Tables

**Table 1:** Proximate composition of raw materials.

%	Wheat flour	Quinoa flour	Amaranth flour
Moisture	11.38 ± 0.09^c^	12.41 ± 0.04^b^	12.62 ± 0.04^a^
Protein	12.54 ± 0.10^b^	17.02 ± 0.10^a^	17.51 ± 0.50^a^
Fat	1.00 ± 0.02^c^	6.60 ± 0.20^a^	6.54 ± 0.02^b^
Ash	0.58 ± 0.01^c^	2.65 ± 0.04^b^	2.82 ± 0.05^a^
Fiber	3.90 ± 0.10^b^	15.36 ± 2.90^a^	14.20 ± 0.60^a^

The mean ± SD (standard deviation) within rows with different small letters differs significantly (*p* < 0.05).

**Table 2:** Chemical analysis of the gluten-free cake.

Sample	Moisture (%)	Protein (%)	Fat (%)	Ash (%)	Fiber (%)
C	41.08 ± 1.27^e^	3.44 ± 0.13^d^	6.37 ± 0.08^d^	1.26 ± 1.27^c^	15.22 ± 0.90^d^
A10	41.51 ± 1.09^e^	3.97 ± 0.18^bc^	7.68 ± 0.11^bc^	1.53 ± 0.04^ab^	17.36 ± 0.11^c^
A20	43.94 ± 2.03^d^	4.14 ± 0.08^b^	7.98 ± 0.41^b^	1.61 ± 1.04^ab^	18.11 ± 0.55^b^
A30	43.91 ± 1.11^d^	4.34 ± 0.24^a^	7.11 ± 0.43^bc^	1.65 ± 1.04^a^	18.91 ± 0.25^a^
Q10	45.04 ± 0.89^c^	3.70 ± 0.06^c^	7.01 ± 0.04^cd^	1.45 ± 1.14^b^	17.27 ± 0.21^c^
Q20	46.54 ± 1.19^b^	3.70 ± 0.09^c^	6.55 ± 0.12^c^	1.47 ± 0.04^b^	18.21 ± 0.15^b^
Q30	47.37 ± 0.99^a^	4.37 ± 0.33^a^	8.69 ± 0.56^a^	1.67 ± 0.04^a^	18.76 ± 0.25^a^

The mean ± SD (standard deviation) within columns with different small letters differs significantly (*p* < 0.05). C: control; A10: sample containing 10% amaranth flour; A20: sample containing 20% amaranth flour; A30: sample containing 30% amaranth flour; Q10: sample containing 10% quinoa flour; A20: sample containing 20% quinoa flour; A30: sample containing 30% quinoa flour.

**Table 3:** Physical analysis of the gluten-free cake.

Sample	Specific volume (cm^3^/g)	Hardness (g)	*L* ^∗^	*a* ^∗^	*b* ^∗^
C	2.55 ± 0.02^bc^	470.89 ± 13.06^b^	79 ± 1.54^a^	0.29 ± 0.11^c^	19.16 ± 0.35^d^
A10	2.58 ± 0.07^bc^	396.01 ± 13.18^bc^	76.98 ± 1.78^b^	1.08 ± 0.44^ab^	21.91 ± 1.78^c^
A20	2.30 ± 0.07^c^	394.76 ± 11.08^c^	75.25 ± 1.31^bc^	1.12 ± 0.11^ab^	21.65 ± 1.25^c^
A30	2.65 ± 1.11^b^	546.34 ± 10.24^ab^	74.11 ± 0.80^cd^	1.91 ± 0.44^a^	24.20 ± 0.75^a^
Q10	2.36 ± 0.06^c^	568.97 ± 11.08^a^	76.51 ± 1.24^b^	0.45 ± 0.20^b^	21.91 ± 1.01^c^
Q20	2.88 ± 0.09^a^	259.33 ± 10.09^d^	73.45 ± 1.47^c^	0.63 ± 0.24^b^	22.38 ± 0.95^b^
Q30	2.67 ± 0.03^b^	447.37 ± 13.33^bc^	72.09 ± 1.26^d^	1.98 ± 0.36^a^	24.37 ± 1.21^a^

The mean ± SD (standard deviation) within columns with different small letters differs significantly (*p* < 0.05). C: control; A10: sample containing 10% amaranth flour; A20: sample containing 20% amaranth flour; A30: sample containing 30% amaranth flour; Q10: sample containing 10% quinoa flour; A20: sample containing 20% quinoa flour; A30: sample containing 30% quinoa flour.

**Table 4:** Fatty acid and mineral composition of the formulated gluten-free cake.

Nutrients	Wheat cake	Quinoa cake	Amaranth cake
Oleic acid (%)	0.95	1.2	1.7
Linoleic acid (%)	1.5	1.6	2.7
Linolenic acid (%)	0.25	0.32	0.37
Iron (mg/kg)	0.69	110	2.5
Calcium (mg/kg)	0.48	20.3	23.5
Magnesium (mg/kg)	1.97	82	54
Zinc (mg/kg)	0.65	35.8	1.9

## Data Availability

The data used to support the findings of this study are available from the corresponding author upon request.
